# In Vitro Aging of Human Skin Fibroblasts: Age-Dependent Changes in 4-Hydroxynonenal Metabolism

**DOI:** 10.3390/antiox9020150

**Published:** 2020-02-11

**Authors:** Igor Petkovic, Nikolaus Bresgen, Ettore Gilardoni, Luca Regazzoni, Koji Uchida, Giancarlo Aldini, Werner Siems, Peter Eckl

**Affiliations:** 1 Department of Biosciences, University of Salzburg, Hellbrunnerstrasse 34, 5020 Salzburg, Austria; nikolaus.bresgen@sbg.ac.at (N.B.); igorpetko@yahoo.com (P.E.); 2 Department of Pharmaceutical Sciences, Università degli Studi di Milano, Via Mangiagalli 25, 20133 Milan, Italy; ettore.gilardoni@unimi.it (E.G.); luca.regazzoni@unimi.it (L.R.); giancarlo.aldini@unimi.it (G.A.); 3 Graduate School of Agricultural and Life Sciences, University of Tokyo, Tokyo 113-8657, Japan; a-uchida@mail.ecc.u-tokyo.ac.jp; 4 Clinics for Prevention and Rehabilitation, Burgstrasse 35, 38667 Bad Harzburg, Germany; werner.siems@t-online.de

**Keywords:** oxidative stress, lipid peroxidation, dermal fibroblasts, aging, 4-hydroxynonenal

## Abstract

Evidence suggests that the increased production of free radicals and reactive oxygen species lead to cellular aging. One of the consequences is lipid peroxidation generating reactive aldehydic products, such as 4-hydroxynonenal (HNE) that modify proteins and form adducts with DNA bases. To prevent damage by HNE, it is metabolized. The primary metabolic products are the glutathione conjugate (GSH-HNE), the corresponding 4-hydroxynonenoic acid (HNA), and the alcohol 1,4-dihydroxynonene (DHN). Since HNE metabolism can potentially change during in vitro aging, cell cultures of primary human dermal fibroblasts from several donors were cultured until senescence. After different time points up to 30 min of incubation with 5 µM HNE, the extracellular medium was analyzed for metabolites via liquid chromatography coupled with electrospray ionization mass spectrometry (LC/ESI-MS). The metabolites appeared in the extracellular medium 5 min after incubation followed by a time-dependent increase. But, the formation of GSH-HNL and GSH-DHN decreased with increasing in vitro age. As a consequence, the HNE levels in the cells increase and there is more protein modification observed. Furthermore, after 3 h of incubation with 5 µM HNE, younger cells showed less proliferative capacity, while in older cells slight increase in the mitotic index was noticed.

## 1. Introduction

Aging is defined as the accumulation of deleterious changes in cells over time, which increase the risk of disease and ultimately lead to death. Over decades, many theories have emerged and proven that aging is a multifactorial process. The evolutionary theory of aging considers the declining force of natural selection as a cause of aging [1]. The gene regulation theory of aging suggests that changes in the expression of certain genes cause aging. This theory is still controversial, although many genes exhibit different expression pattern with age [2]. Furthermore, telomere shortening and aging are closely related because the length of telomeres decreases with every cell division leading to replicative and cellular senescence [3]. The neuroendocrine theory proposes that aging is caused by the failure of neurological and endocrine regulation of cell function [4]. This theory and the immune theory of aging are cross-linked through neuropeptides and cytokines that maintain communication between the neuroendocrine and the immune system [5]. The free radical theory explains aging as a consequence of cumulative oxidative damage caused by endogenous free radicals [6]. This theory has been criticized in the past because there was no experimental support that free radicals exist in the living organism. However, after work on electron paramagnetic resonance and the identification of the hydroxyl radical, and particularly after the enzyme superoxide dismutase (SOD) was discovered, this theory had scientific proof that free radicals can be generated endogenously [7,8].

Increased production of reactive oxygen species (ROS) in aging is associated with increased damage to biomolecules [9]. The accumulation of oxidative damage leads to the emergence of various pathophysiological conditions, including age-related neurodegenerative diseases [10,11]. ROS cause damage to lipids, leading to their oxidative deterioration and production of α,β-unsaturated aldehydes [12,13]. 4-Hydroxynonenal (HNE) is a major breakdown product of ω-6-polyunsaturated fatty acids (e.g., linoleic, 18:2 and arachidonic acid, 20:4), and it has been reported to be a cytotoxic and genotoxic molecule [14,15,16]. Unlike free radicals which react only with biomolecules in their vicinity due to their short half-life, byproducts of lipid peroxidation are more stable and react with target molecules that are far from the place of their origin. As with the other reactive species, proteins react with HNE and form adducts eventually impairing protein function. Amino and sulphydryl groups of cysteine, histidine, and lysine residues are the most reactive with HNE. Nevertheless, protein residues are important for HNE-protein formation and their deleterious effects, as well as protein structure and whether targeted amino acids are important for protein function. Given that proteins are abundantly present in cells, adduct formation leads to changes of the tertiary protein structure, inhibition of enzyme activity, as well as changes in gene regulation and cell signaling. Therefore, HNE-protein adducts are good biomarkers for oxidative stress [17]. In order to prevent the accumulation of HNE and, consequently, the modification of biomolecules, tissue damage, and disease progression, cells develop mechanisms to rapidly metabolize HNE. HNE metabolism has been studied in different cell types and the major metabolites found are adducts of glutathione (GSH) and HNE (GSH-HNE), and the metabolites generated by the oxidation and reduction of the carbonyl group of HNE, resulting in the corresponding 4-hydroxynonenoic acid (HNA) and alcohol 1,4-dihyroxynonene (DHN) [18]. Glutathione-S-transferase (GST), especially the alpha class GSTA4-4, is very specific towards HNE and catalyzes the formation of GSH-HNE [19]. HNA and DHN are the result of HNE oxidation and reduction by aldehyde dehydrogenase (ALDH) and alcohol dehydrogenase (ADH), respectively. Additionally, GSH-HNE in the reaction catalyzed by ADH and ALDH results in GHS-DHN and GSH-HNA. The latter further undergoes intramolecular rearrangement generating the corresponding cyclic lactone (GSH-HNL) [20].

HNE metabolism serves as the cell’s secondary antioxidant defense. Therefore, this study addressed whether an aging cell loses the ability to metabolize HNE efficiently. This was analyzed via the characterization of 4-HNE adducts with glutathione in human dermal fibroblasts, which serve as a good model for in vitro aging [21]. The understanding of in vitro aging provides valuable information about the aging process in vivo, as well as comprehension of the mechanisms involved in various degenerative processes accompanying aging. During the process of in vitro, aging cells undergo changes in morphology. The cells in later passages are bigger and their morphology is not as homogenous as in the first passages. They become flattened, accumulate debris, and show altered gene expression [22]. The time point when a cell becomes senescent and the number of cell doublings depends on the cell type, donor age, and genotype [23], for example, a reverse correlation between passage number and donor age is described [24]. Replicative or cellular senescence can be triggered by telomere shortening, oxidative stress induced DNA damage, chromatin disorder, and oncogene induction [25]. Depending on its concentration, HNE affects cell proliferation, transformation, and cell death [26]. This study, therefore, also aimed to elaborate whether the cells at different passages react differently to sublethal 4-HNE concentrations with respect to cell proliferation and apoptosis.

## 2. Materials and Methods

### 2.1. Chemicals

Serum free minimum essential medium (MEM), collagenase ll, dispase ll, and fetal bovine serum (FBS) were obtained from Gibco, Vienna, Austria. Penicillin and streptomycin were from PAA Laboratories, Pasching, Austria. Trypsin/EDTA was obtained from Lonza via Szabo-Scandic, Vienna, Austria. Nonessential amino acids, sodium pyruvate, aspartate, L-serine, and all other chemicals for cell culture, unless otherwise specified, were obtained from Sigma-Aldrich, Vienna, Austria. Nonfat dry milk was obtained from Bio-Rad laboratories, Herculaes, CA, USA. HRP-conjugated secondary antibody (goat anti-mouse IgG) was purchased from Abcam, UK. Nitrocellulose membrane was obtained from Thermo Fisher Scientific, Rockford, Illinois, USA. Plastic labware was purchased from Greiner, Vienna, Austria. HPLC grade water (18MΩ) was produced with a Milli-Q water system (Millipore; Milan; Italy). HPLC grade solvents, trifluoracetic acid (TFA), PBS, GSH, and formic acid (FA) were purchased from Sigma-Aldrich, Milan, Italy.

Cell culture MEM was supplemented with 0.2 mM aspartate, 0.2 mM serine, 10 mM pyruvate, 10% FBS, and 1% penicillin-streptomycin (100 U/mL).

### 2.2. Cell Culture

Fibroblasts were isolated by enzymatic tissue dissociation from foreskins of four healthy male donors. The tissue samples were obtained from Paracelsus Medical University Clinics Salzburg, and tissue collection was approved by the Ethics Committee of the University of Salzburg (ref. no. 415-EP/73/548-2015). Initially, tissues were washed in 70% ethanol and rinsed with PBS. The tissues were minced into small pieces and incubated overnight at 4 °C with a few ml of dispase II (2.24 U/mL) solution, diluted 1:1 in PBS followed by additional two to four hours incubation in a cell culture incubator at 37 °C, 95% relative humidity, and 5% CO2. Dispase ll enables separation of the dermis from the epidermal layer. The dermal layer was incubated overnight with collagenase II (100 U/mL), diluted in PBS and, then, suspended by gentle shaking. The suspension was filtered through a sterile nylon filter (40 µm mesh width) and the filter was washed with MEM. The cell suspension was centrifuged at 1000 rpm for 10 min, the supernatant was discarded, and the cells were resuspended in complete medium. Cells were seeded into a T-75 cell culture flask and maintained and grown to confluence in the incubator with a medium change every third day. When cells were approximately 80% confluent, the cells were detached with 0.25% trypsin-EDTA and passaged (splitting ratio 1:3).

### 2.3. Western Blot Analysis

At every sixth passage, 1 × 10^6^ fibroblasts were harvested, centrifuged for 10 min at 1000 rpm, and lysed with ice-cold cell lysis buffer (50 mM Tris-HCl (pH 8), 150 mM NaCl, 1% Triton X-100, 1 mM EDTA) in the presence of protease inhibitor cocktail (Roche). The lysate was refined by centrifugation for 15 min at 14,000 rpm, at 4 °C and the supernatants were used for the protein concentration quantification by using the Bradford assay [27]. Then, 40 µg of proteins, diluted in 4× Laemmli sample buffer (Bio-Rad), for each pocket of the gel were separated by sodium dodecyl sulfate polyacrylamide gel electrophoresis (SDS-PAGE) using stacking (4%, pH 6.8) and resolving gel (12%, pH 8.8). Thereafter, proteins were electro-transferred to a nitrocellulose membrane. To prevent nonspecific binding of the antibody to the membrane, the membrane was blocked with blocking buffer (5% nonfat dry milk diluted in tris-buffered saline Tween 20 (TBST)). Protein immunodetection was performed using primary mouse-anti-HNE-his monoclonal antibody (developed in the Koji Uchida’s lab, Tokyo University, Tokyo, Japan) diluted 1:2000 and HRP-conjugated secondary goat-anti-mouse antibody diluted 1:10000 in blocking buffer. Proteins were detected by chemiluminescence detection system (FUSION, Vilber Lourmat, Eberhardzell, Germany). Obtained images and band densities were analyzed by the software provided with the FUSION detection system.

### 2.4. Incubation with HNE

**Figure 1:**
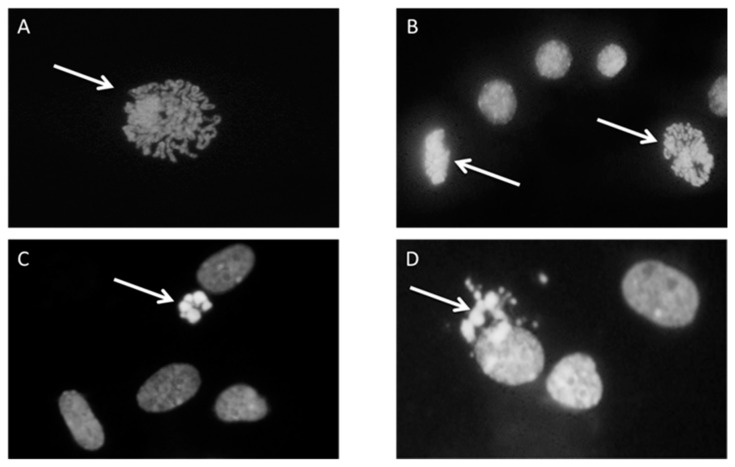
Cell proliferation and apoptosis, cells were stained with 4’,6-diamidino-2-phenylindole (DAPI) and pictures were taken under fluorescence microscope. Arrows represent the cell in the state of (**A**) Prophase; (**B**) prophase and metaphase; (**C**) early apoptosis; (**D**) late apoptosis.

First, 1 × 10^6^ cells were plated in a 60 mm diameter Petri dish with serum-free MEM and incubated with 5 µM HNE for 3 h in a cell culture incubator at 37 °C, 95% relative humidity, and 5% CO_2_. After incubation under standard culture conditions, cells were left to grow for an additional 24 h in a medium with 10% FBS. The second Petri dish was incubated under the same conditions and served as control. The experiment was run in two experimental replicates. For mitotic and apoptotic index determination, cells were fixed with 5 mL methanol/glacial acetic acid (3:1) for 15 min followed by washing with distilled water for 2 min and air drying. The fixed cells were washed with McIlvaine buffer (0.2 M Na2HPO4, 0.1 M citric acid, pH 7) and stained with a 4’,6-diamidino-2-phenylindole (DAPI) solution (0.2 µg/mL) for 1 h, in the dark. After incubation with DAPI, cells were washed again with McIlvain and, briefly, with distilled water and left to dry. Stained cells were covered with glycerol and a thousand cells per dish were analyzed under the fluorescence microscope (Leitz, Aristoplan) to determine the number of mitotic and apoptotic cells. The assessment was based on the morphology of stained nuclei (mitotic cells in different stages of mitosis, and apoptotic cells with condensed and fragmented nucleus) (Figure 1).

At every third passage, fibroblasts were trypsinized and counted using a hemocytometer. Then, 1 × 10^6^ cells were plated in a 60 mm diameter Petri dish with MEM containing 10% FBS and 1% antibiotics. After attachment, cells were washed twice with pre-warmed PBS which had been replaced by serum-free MEM, and 100 µL were taken as a control sample and, then, challenged with 5 µM HNE for 30 min. The HNE was prepared from 4-hydroxynonenal-diethylacetate (HNE-DEA) by hydrolysis with 1 mM HCl, 1 h at room temperature, and calculated from the absorption at λmax 224 nm and the molar extinction coefficient ε = 13750 L mol^−1^ cm^−1^. During the 30 min of HNE treatment, 100 µL of medium each were taken at different time intervals (15 s, 1, 2, 5, 10, 20, and 30 min), transferred into cryovials, and stored at –80 °C, until further use to compare the kinetics of HNE metabolism in the cells in the earlier and later passages of cell culture by using liquid chromatography coupled with electrospray ionization mass spectrometry (LC/ESI-MS).

### 2.5. Synthesis of the Glutathione-HNE Adduct

HNE or deuterated HNE (HNEd5) was obtained by hydrolysis of the corresponding HNE-dimethylacetal (HNE-DMA) in 1 mM HCl for 1 h at room temperature. HNE-DMA was synthetized as reported by Rees and colleagues [28]. Glutathione-HNE and GSH-HNEd5 adducts were synthesized mixing glutathione and HNE or HNEd5 2:1 for 3 h at 37 °C, in 10 mM PBS. Adduct formation was monitored by HPLC-UV analysis following the chromatographic peak corresponding to HNE extrapolated at λmax = 225 nm. Briefly, an aliquot of the reaction was diluted and acidified to obtain a final concentration of 40 mM HCl in 90% H2O and 10% ACN. Then, 80 µL of the solution were injected by partial loop mode into the platform consisting of a Surveyor HPLC system (Thermo Scientific, Rodano, MI, Italy) made up of a MS quaternary pump at a flow rate of 300 µL/min for all the experiments. Eluents made up of 1 mM HCl (eluent A) and ACN (eluent B) were used. The analyte was separated with a Phenomenex Kinetex C18 column (25 mm × 2.10 mm, 2.6 µm, 100 Å). Adducts were stored at −20 °C until use.

### 2.6. LC/ESI-MS Analysis

Before LC-MS analysis, extracellular medium samples were diluted 1:3 with 1% trifluoroacetic acid (TFA) and then spiked with the internal standard at a concentration of 1 µM. The samples were ready for autosampler injection after centrifugation at 14,000 rpm, for 10 min. The analytical platform consisted of an Ultimate 3000 RSLCnano system coupled with an LTQ-Orbitrap XL mass spectrometer through a Finnigan IonMax electrospray ionization source (ESI) (Thermo Scientific, Rodano, MI, Italy). Ionization was performed in positive ion mode with spray voltage 3.5 kV, capillary temperature 220 °C, and capillary voltage 35 kV. During the analysis, the MS spectra were acquired in profile mode by Orbitrap using the following settings: Scan range 250 to 700 *m/z*, 5 × 105 ions per scan, maximum injection time was set to 500 ms, and resolution was set to 100,000 (FWHM at *m/z* 400). Lock mass option was enabled to provide a real-time internal mass calibration during the analysis using a reference list of 20 abundant and known background signals, already reported by Keller et al. [29] as common air contaminants in mass spectrometry. Instrument control was provided by the software Xcalibur 2.0 and Chromeleon Xpress 6.8 (Thermo Scientific, Rodano, MI, Italy).

First, 15 µL of a sample were loaded, with partial loop injection, on a µ-Precolumn Cartridge (PrepMap100 C18, 5 µm, 100 Å, 300 µm i.d. × 5 mm, Dionex) for sample clean up and preconcentration for three minutes at a flow rate of 10 µl/min, using 99% mobile phase A (0.1% aqueous TFA) and 1% mobile phase B (0.1% HCOOH diluted in acetonitrile). Then, the precolumn was diverted online to a Hypersil Gold Capillary Column (C18, 5 µm, 0.18 mm i.d. × 100 mm 175 Å, Thermo Fischer Scientific) as an analytical column for metabolite separation. The analytes were eluted applying a 30 min ramp gradient using solvents A 0.1% aqueous HCOOH and B 0.1% HCOOH diluted in acetonitrile, at a flow rate of 1.5 µL/min. Three minutes after loading, the solvent B percentage was increased from 5% to 95% within 18 min and kept constant for four minutes, followed by equilibration for five minutes at the initial conditions. The injection was done in partial loop mode. The glutathione adducts eluted with a retention time of 14.1 min for glutathione-HNE (GSH-HNE) and deuterated GSH-HNE (GSH-dHNE), 13.9 min for glutathione-dihydroxynonene (GSH-DHN) and 15.7 min for the corresponding cyclic lactone of glutathione-hydroxynonenoic acid (GSH-HNL).

**Figure 2:**
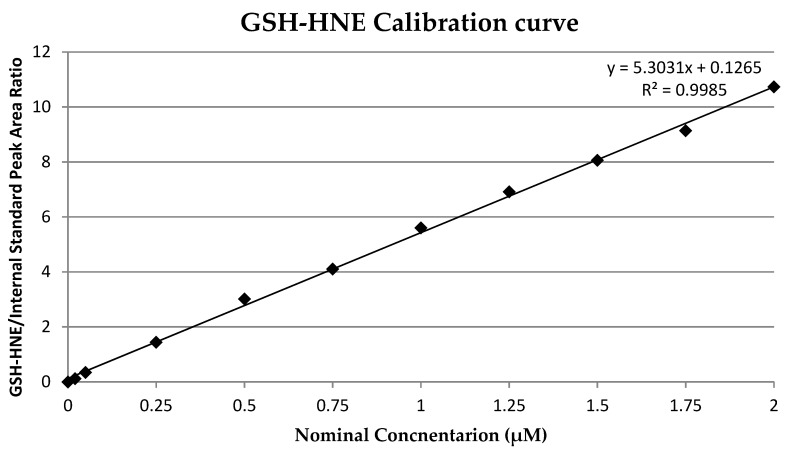
Glutathion and 4-hydroxynonenal (GSH-HNE) calibration curve.

A calibration curve was prepared by spiking pure medium with adduct solution to give concentrations of 0.02, 0.05, 0.25, 0.5, 1, 1.25, 1.5, 1.75, and 2 µM. Three independent samples were prepared for each concentration. The calibration curve (Figure 2) was calculated by least square linear regression analysis of the nominal concentration of adduct versus adduct/internal standard peak area (*R^2^* = 0.9985). The limit of detection (LOD) and limit of quantification (LOQ) were determined as 0.02 µM and 0.05 µM, respectively. As an internal standard, deuterated GSH-HNE was used at a final concentration of 1 µM.

### 2.7. Statistical Analysis

The normality of data distribution was checked applying the Shapiro–Wilks test. For the determination of the significance levels for changes of the mitotic and apoptotic index, as well as differences in HNE metabolism between passages, Student’s two-tailed t-test for independent samples was applied. Analyses were performed using SPSS version 20 (IBM SPSS Statistics) and GrpahPad Prism version 8 (GraphPad Software). Results were considered statistically significant when the *p*–value was <0.05. 

## 3. Results

**Figure 3:**
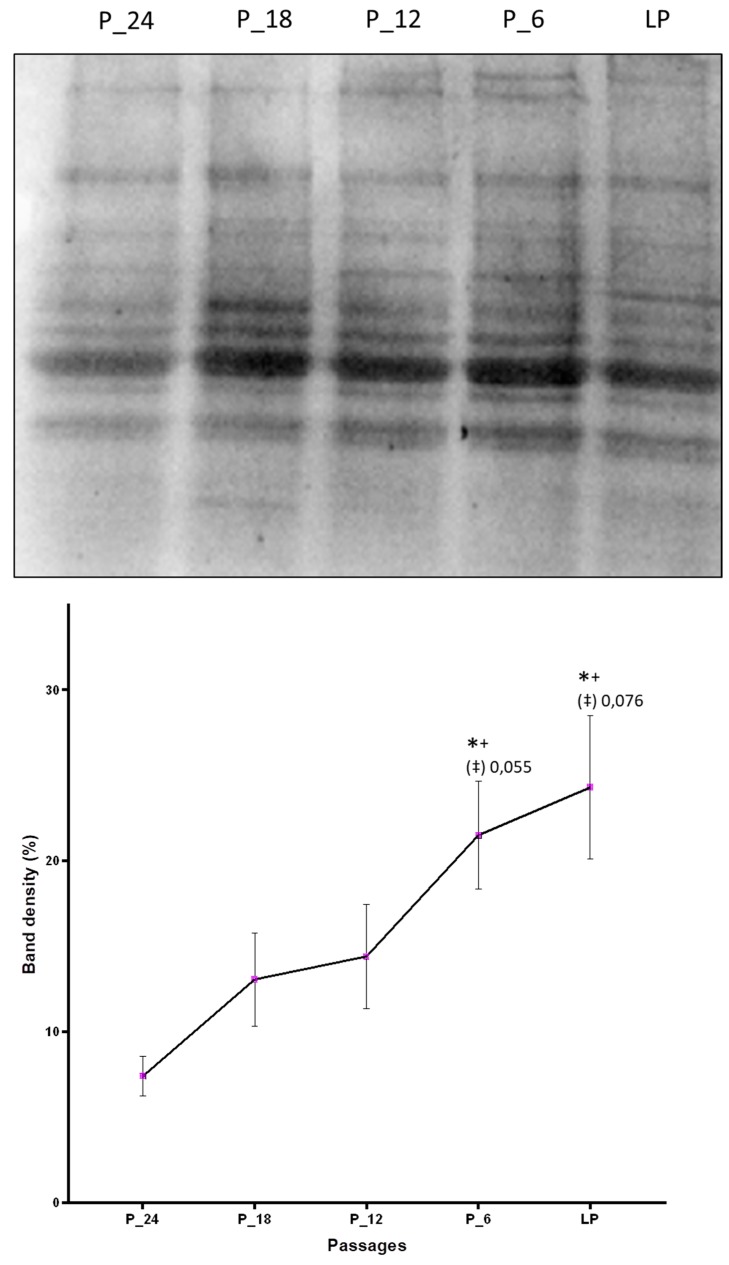
Changes of the amount of HNE-modified proteins due to in vitro aging assessed by Western blot analysis applying anti-HNE-his monoclonal antibodies. The band intensities were quantified by densitometry. LP indicates last passage. Data are presented as mean ± SD of band density expressed in percentages. Statistically significant differences are marked with * as compared with P-24, with + as compared with P-18, and with ‡ as compared with P-12, for *p* < 0.05, as analyzed by Student’s two-tailed t-test for independent samples (*n* = 4).

By Western blot analysis it was confirmed that aging cells accumulate more HNE-modified proteins as compared with the cells on the onset of the cell culture. Taking into account five passages (P-24, P-18, P-12, P-6, and last passage) that are common to the fibroblast cell culture of all four donors in which protein modification was studied, a constant increase of the amount of HNE-modified proteins with increasing number of passages was observed, with statistically significant differences between the last passage and the first measurement point, P-24 (Figure 3).

**Figure 4:**
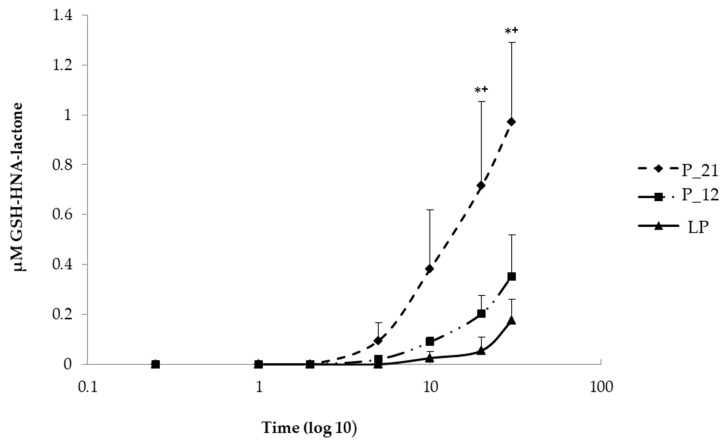
Comparison of the kinetics of GSH, 4-hydroxynonenoic acid, and lactone (GSH-HNA-lactone) formation at early (P-21), middle (P-12), and last passage (LP) in extracellular media taken in different time points (0, 15 s, 1 min, 2 min, 5 min, 10 min, 20 min, and 30 min) after the addition of HNE. Data are presented as means ± SD (P-21 and P-12 *n* = 4, LP *n* = 3). Statistically significant differences are marked with * *p* < 0.05 when P-21 is compared with LP and +*p* < 0.05 when P-21 is compared with P-12 as analyzed by Student’s two-tailed t-test for independent samples.

To understand the increased rate of HNE-modified proteins, the cells were challenged with 5 µM HNE for 30 min in order to determine if this is caused by the impaired HNE metabolism. For this purpose, 100 µL each of extracellular medium were taken at different time intervals (15 s, 1, 2, 5, 10, 20, and 30 min) after HNE addition and analyzed by LC/ESI-MS. The metabolism was monitored via the formation of two GSH-HNE metabolites, GSH-HNL and GSH-DHN. GSH-HNE, as such, was difficult to identify because likely the oxidation and reduction to the corresponding alcohol and acid occurred immediately in the cell. By comparing the GSH-HNL kinetics, there was a difference in earlier and later passages of cell culture, at the last, and the 12th, and the 21st passages before senescence, which are defined as late, middle, and early passages, respectively, GSH-HNL was not detectable until five minutes after the addition of the HNE. After five minutes, around 90 nM GSH-HNL was detected in the early passage (P-21). After 10 min, four times as much GSH-HNL was formed in passage P-21 (~400 nM), while at the last passage an increase in the concentration of only a few nM was found. After 20 min of incubation with 5 µM HNE, there was a significant increase of the GSH-HNL concentration in passage P-21 (~700 nM) as compared with the last passage (~90 nM). Analysis of extracellular medium further demonstrated that a concentration of around 1 µM of GSH-HNL could be verified at the early passage and about 200 nM at the last passage after 30 min of incubation. These represent 20% and 4% of the initial HNE concentration, respectively (Figure 4).

**Figure 5:**
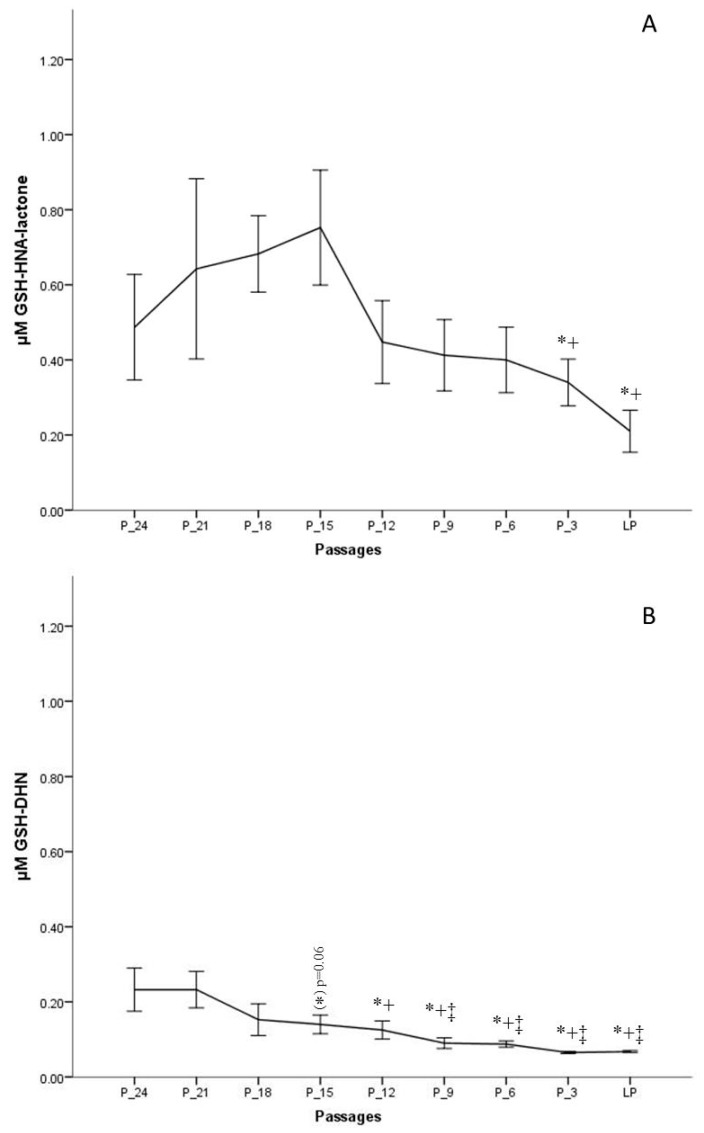
Concentrations of GSH-HNA-lactone (**A**) and GSH and 1,4-dihydroxynonene (GSH-DHN) (**B**) conjugates measured after 30 min of incubation with 5 µM HNE at different passages of fibroblast culture. Data are presented as means ± SD (*n* = 4). (**A**) Statistically significant differences are marked with * as compared with P-18 and with + as compared with P-15. (**B**) Statistically significant differences are marked with ‡ as compared with P-24, * as compared with P-21, and with + as compared with P-15; for *p* < 0.05, as analyzed by Student’s two-tailed t-test for independent samples.

The results indicate that the formation of GSH-HNL differs depending on the passage number. To obtain a more realistic view on changes of HNE metabolism during in vitro aging, the last passage of every donor was taken as the endpoint, from which data were gathered backwards, for example, P-3 means three passages backwards. This method of presentation was chosen since the donor age differed significantly, from two to 48 years, and would lead to misinterpretations. Figure 5A clearly shows that the concentrations from the onset of the cell culture to 15 passages before senescence is increasing. The highest metabolite concentration after 30 min of incubation was around 1 µM, measured 15 passages before senescence, which is about 20% of the initially added HNE concentration. After that point, as from passage 12 before senescence, there is a continuous decrease of metabolite formation. 

A further metabolite analyzed, in this study, was GSH-DHN, which is formed by the reduction of GSH-HNE by an alcohol dehydrogenase (aldose reductase). GSH-DHN was also found to be released from fibroblasts into the extracellular medium in a time-dependent manner. However, a smaller amount of this metabolite was formed as compared with the lactone. The highest measured concentration, 24 passages before senescence, was ~220 nM, and was followed by a steady decrease. On the basis of the measured concentrations of GSH-DHN, it is assumed that less than 5% of the amount of HNE added is metabolized via the formation of the GSH-DHN adduct in earlier passages, while the concentration found in medium at late passages was not more than 2% (~70 nM) (Figure 5B).

**Figure 6:**
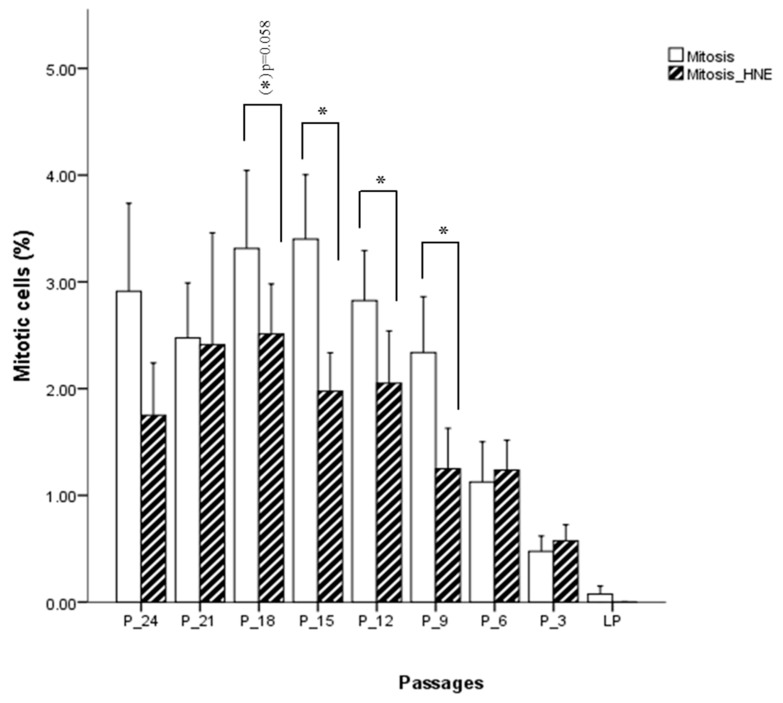
Influence of HNE treatment on fibroblast cell proliferation at different passages of cell culture. Data are presented as means ± SD of the percentage of mitotic cells in culture (*n* = 4). Statistically significant differences are marked with * for *p* < 0.05 as analyzed by Student’s two-tailed t-test for independent samples.

Fibroblasts incubated for 3 h with 5 µM HNE were morphologically analyzed, and no phenotypical difference could be detected between treated and untreated cells. At the onset of cell culture, around 3% of the cells were in M-phase. As expected, the percentage of cells in M-phase decreased when cells were approaching the senescent state. Figure 6 shows that proliferation is lower in the cells treated with 5 µM HNE relative to untreated cells. This is seen up to six passages before senescence was reached, with statistically significant differences at the 18th, 15th, 12th, and 9th passage before senescence (p < 0.05), after which proliferation was slightly higher in the treated cells as compared with the control but not statistically significant. The analysis of apoptotic cell death (from a thousand counted cells) revealed that only 0.05% to 0.2% of the cells were in the stage of apoptosis. Their number increased slightly when cells were approaching the state of senescence. HNE treated cells showed increased levels of apoptosis in almost all passages. However, there is no clear indication whether the cells, depending on their age, respond differently to the treatment of HNE. Interestingly, at the beginning of cell culture, a significantly larger number of apoptotic cells were observed in the HNE-treated cells as compared with the controls.

## 4. Discussion

The focus of this investigation was lipid peroxidation, the most prominent feature of oxidative stress. The emphasis was on 4-hydroxynonenal, the major and the most toxic α,β-unsaturated aldehyde generated during the oxidative breakdown of polyunsaturated fatty acids. Given that HNE affects cell proliferation, transformation, and cell death in a concentration dependent manner [15,18,30], the intention was to elaborate whether a sublethal concentration (5 µM) of 4-HNE has an impact on the proliferative capacity of fibroblasts during in vitro aging. On the one hand, low concentrations of 4-HNE (1 to 2.5 µM) promote cell proliferation as demonstrated in a study of Ruef et al. (1998) with rat smooth muscle cells [31]. However, on the other hand, according to Halliwell and Gutteridge (2007), HNE concentrations which are achieved in the state of oxidative stress (2 to 20 µM) are toxic to many cells and inhibit cell proliferation [26]. This was also confirmed with cultured Ehrlich ascites tumor cells treated with 10 to 20 µM HNE [32], and even lower concentrations of HNE (0.1 µM), that provoked genotoxic effects in rat hepatocytes, as evidenced via the analysis of sister chromatid exchanges [33]. 

This study revealed that a concentration of 5 µM HNE inhibits cell proliferation up to six passages before senescence, but there is a trend towards the induction of cell growth in later passages. This could be explainable by the fact that younger cells have mechanisms to neutralize added HNE via rapid metabolism and conjugation with glutathione. However, in the cells that approach the state of senescence, the metabolic capacity is reduced and the level of free radicals and lipid peroxidation products, such as HNE, is already increased causing a reduction of the GSH pool. Under these circumstances, increased proliferation reduces the intracellular concentration of both HNE and HNE-modified proteins [34].

Higher levels of HNE and prolonged oxidative stress exposure compromise cellular functions, and eventually lead to cell death. In our study, at all examined passages, except at the last, 5 μM HNE caused an increase in the number of apoptotic cells as compared with the non-treated controls, but without a clear age dependence. The increased rate of apoptoses upon HNE treatment suggests that even sublethal concentrations of HNE induce cell death. Investigations on human retinal pigment epithelial cells have proven that HNE causes apoptosis by increasing the expression of the tumor suppressor protein 53 [35]. The involvement of the p53 apoptotic pathway has also been confirmed in other cell lines, for example, the increased expression of p53 in aging Purkinje cells could be the reason for the death of these cells [36,37]. Furthermore, Uchida et al. (1999) reported that HNE via the activation of c-Jun N-terminal kinase (JNK) induced c-Jun expression which leads to upregulation of proapoptotic AP-1 dependent genes such as FasL [38]. This assumption was confirmed in c-Jun deficient mouse fibroblasts which were resistant to cell death accompanied by FasL downregulation [39]. However, an earlier publication pointed out that c-Jun deficient mouse fibroblasts show a decline of proliferation with elevated levels of p53 [40]. Therefore, additional work should be carried out to elaborate the role of HNE in the aging process. 

Previous studies with various cell types have shown that 2% to 8% of cellular proteins are modified after the addition of HNE [41]. HNE adduction generates misfolded and damaged proteins with impaired function [42], particularly enzymes when HNE reacts with the active site amino acids. The content of oxidatively modified proteins has been found to increase in cultured fibroblasts, with age above 60 [43]. Therefore, we wanted to analyze the rate of protein HNE modification in human skin fibroblast cells undergoing in vitro aging. The results of Western blot analysis, using an anti-HNE-his antibody, show that fibroblasts in later passages have more HNE modified proteins as compared with early passages, where modified proteins were also detected. Our results are in agreement with previous studies on aging embryonic lung and facial skin fibroblasts [44,45] but also in keratinocytes [46]. The reason for elevated levels of HNE modified proteins in aging cells could be an increase in the rate of HNE production, an impaired antioxidant defence, or impaired HNE metabolism, and decrease of proteasome activity [46,47]. HNE as a bifunctional molecule is able to form cross-linked proteins, which cannot be degraded by the proteasome. Furthermore, the HNE cross-linked proteins are able to inhibit the proteasome [48,49,50]. HNE modified proteins, their aggregates, and HNE cross-linked proteins are found in many age-related neurodegenerative pathologies, for example, Alzheimer’s disease, Parkinson disease, Huntington disease, and ALS [51,52]. Therefore, comprehension of the mechanism of aldehyde adduction to proteins and their degradation by cellular repair systems gives us a tool to better understand the pathologies in which these modifications are involved.

The accumulation of modified proteins in aging could be dependent on the intracellular rate of HNE metabolism. The enzymatic metabolism of HNE goes through two phases. Phase l includes HNE reduction and oxidation resulting in the corresponding alcohol and the carboxylic acid. Phase ll is accomplished by Michael addition of GSH to HNE. The GSH-HNE conjugate immediately undergoes reduction to GSH-DHN or oxidation to GSH-HNA, which can further form a cyclic lactone by intramolecular rearrangement [20]. Therefore, these two metabolites were a further focus of this investigation and the following result was obtained: For GSH-HNL, the metabolite levels increased up to the 15th passage before senescence followed by a steady and significant decrease. In the case of GSH-DHN, the decreasing trend was observed from the beginning of cell culture. This behavior of GSH-HNL formation can be explained by an initial adaptation to increasing endogenous formation of HNE. The following decrease can be interpreted in terms of a loss of the ability to detoxify HNE with an increasing in vitro age. This decline could be due to reduced glutathione concentrations observed in aging cells [53] and a consequent decrease in the formation of GSH-HNE conjugates. Another reason could be an insufficient GSTA4 activity, the main enzyme for Michael addition of GSH to HNE [19]. Nevertheless, whenever the amount of GSH present in the cell is not sufficient, even higher expression of GSTA4 is useless. These results, at least in part, explain the increased HNE-modified proteins concentrations found in older cells [54]. HNE which is not metabolized raises the intracellular HNE pool. Together with the age-dependent increase of the lipid peroxidation rate reported [55], this intracellular HNE pool is further increased, thus, enhancing the interaction with DNA and proteins. 

The velocity of HNE removal from the cell is not the same in different cell types. The most rapid uptake and detoxification have been shown for rat hepatocytes [56], a much slower metabolism in synovial fibroblasts [57], human polymorphonuclear lymphocytes [58], or colonocytes [59]. In this study, the initially applied HNE amount of 5 µM was not detectable after 1 min in the extracellular medium, but GSH-HNL was detected 5 min after HNE addition. In contrast, GSH-DHN was detectable in the medium after 30 min of incubation in a much lower amount as compared with GSH-HNL. Thus, it is concluded that the oxidation of GSH-HNE is a dominant reaction in the cell. RLIP76, a RalA binding protein, is responsible for the transport of GSH-HNE adducts from the cells, and it has been shown that RILP76 plays a major role in the regulation of intracellular 4-HNE concentration [60]. The higher levels of 4-HNE and GSH-HNE were also observed in RLIP76 knockout mice, in a radiation toxicology study, proving that this protein is important in cellular detoxification [61]. Senescence could theoretically also affect exocytotic activity, however, it is not known whether this would also influence RLIP79 based release of GSH-metabolites. In addition to these two metabolites, an investigation by Aldini et al. (2003) with human keratinocytes revealed S-(4-oxononanal-3-yl)-glutathione, the oxidation product of secondary alcohol on C-4, as one of the most abundant metabolites [62]. In the same study it was further shown that more than twice the amount of metabolite was still in the cell 30 min after incubation with 100 µM HNE as compared with the amount in the extracellular medium and the plateau of formation after 60 min. When these findings are extrapolated to our cells, the measured amounts after 30 min would just be half the amount of metabolite formed inside the cell.
